# Pediatric reference values for total psoas muscle area in Korean children and adolescents

**DOI:** 10.3389/fped.2024.1443523

**Published:** 2025-01-17

**Authors:** Jisoo Kim, Mi-Jung Lee, Hyun Ji Lim, Yonghan Kwon, Kyunghwa Han, Haesung Yoon

**Affiliations:** ^1^ Department of Radiology and Research Institute of Radiological Science, Severance Hospital, Yonsei University College of Medicine, Seoul, Republic of Korea; ^2^ Department of Biostatistics and Computing, Yonsei University Graduate School, Seoul, Republic of Korea; ^3^ Department of Radiology, Research Institute of Radiological Science, and Center for Clinical Imaging Data Science, Yonsei University College of Medicine, Seoul, Republic of Korea; ^4^ Department of Radiology, Gangnam Severance Hospital, Yonsei University College of Medicine, Seoul, Republic of Korea

**Keywords:** psoas muscle, sarcopenia, child, adolescent, growth chart

## Abstract

**Background:**

Uniform cut-off values cannot be applied to children because muscle mass changes significantly with growth and different population groups have different races and lifestyles, so reference values from healthy children are needed.

**Materials and methods:**

Pediatric patients (≤18 years of age) who underwent abdominal CT for abdominal pain in the emergency room without a past history from January 2015 to January 2021 were enrolled. Total psoas muscle area (tPMA) was calculated as bilateral psoas muscle areas drawn semi-automatically at the levels of L3–4 and L4–5. Graphs for weight and height were drawn and compared using data from the Korean national growth chart. tPMA distributions according to sex and age were obtained using quantile regression with smoothed polynomials. We analyzed correlations of tPMA with weight and height.

**Results:**

A total of 740 pediatric patients were included [M:F = 340:400, 12 years (interquartile range 9–16)]. When compared to the Korean national growth chart, the 50th percentile values for height and weight in study population were not significantly different. At L3–4, the 50th percentile tPMA values ranged from 416 to 2,802 mm^2^ in males and 370 to 1,501 mm^2^ in females. The 50th percentile tPMA values for L4–5 ranged from 556 to 3,563 mm^2^ in males and from 579 to 2007 mm^2^ in females. In males, tPMA at L3–4 and L4–5 showed positive correlations with weight (*r* = 0.87; *r* = 0.87, *P* < 0.001, both) and height (*r* = 0.82; *r* = 0.84, *P* < 0.001, both). tPMA at L3–4 and L4–5 were positively correlated with weight (*r* = 0.77; *r* = 0.81, *P* < 0.001, both) and height (*r* = 0.61; *r* = 0.67, *P* < 0.001, both) in females.

**Conclusion:**

We provided sex-specific and age-specific growth charts and developed an online calculator for tPMA at the levels of L3–4 and L4–5, which can serve as an evaluation guide for muscle mass in Korean children.

## 1 Introduction

Sarcopenia, a medical condition defined by loss of muscle mass and function, is associated with several adverse outcomes, including loss of motor independence, reduced quality of life and mortality (1, 2). Previous studies have shown the negative clinical impact of sarcopenia through longer hospital stays and post-treatment complications in pediatric patients (3–5).

Quantification methods for diagnosing sarcopenia are dual-energy x-ray absorptiometry (DEXA), bioelectrical impedance analysis, and cross-sectional imaging such as CT or MRI (2). Although no studies have directly examined the correlation between total skeletal muscle mass and total psoas muscle area (tPMA) assessed via CT in pediatric populations, tPMA measured at the lumbar level from a single abdominal CT image has been widely used across various cohorts (6–8). It serves as a valuable quantitative indicator, even in children, as it can be measured more easily and quickly than other markers of muscle mass (9, 10).

In adults, even though a single clear consensus on the definition of sarcopenia using tPMA has not yet been established, some studies have uniformly defined it as muscle mass below the 5th percentile or 2 standard deviations of that of young adults for each sex (2, 11–14). However, a uniform cut-off value cannot be readily applied to children because muscle mass changes greatly with growth. A few studies of healthy Canadian, American and Japanese children have proposed sex-specific and age-specific reference values for muscle mass (6–8). However, different cut-off values have been suggested even when diagnosing sarcopenia in adults, because race and lifestyle such as urbanization can vary both across and within populations, with a past study that analyzed muscle mass using DEXA in the Korean pediatric population observing a different growth pattern from those seen in Caucasians (12, 15, 16).

Therefore, our study aimed to complete growth charts according to sex and age for tPMA at the intervertebral disc levels of L3–4 and L4–5 using CT images taken from healthy Korean children and adolescents who visited the emergency room without any medical history. In addition, we aimed to identify trends of tPMA according to sex and age and provide an online calculator on a separate webpage that could provide a relative percentile for patients of the same sex and age.

## 2 Materials and methods

### 2.1 Subjects

This retrospective study was approved by the Institutional Review Board of the authors' institution. Pediatric patients under the age of 18 who underwent abdominal CT for acute abdominal pain in the emergency room from January 2015 to January 2021 were enrolled. Exclusion criteria were (a) history of previous surgery or other chronic disease, (b) newly discovered underlying chronic disease during this visit, (c) measurement failure due to poor image quality, and (d) missing medical data. Medical records were reviewed to collect clinical data including sex, age, weight, and height.

### 2.2 Image analysis: measurement of tPMA

**Figure 1:**
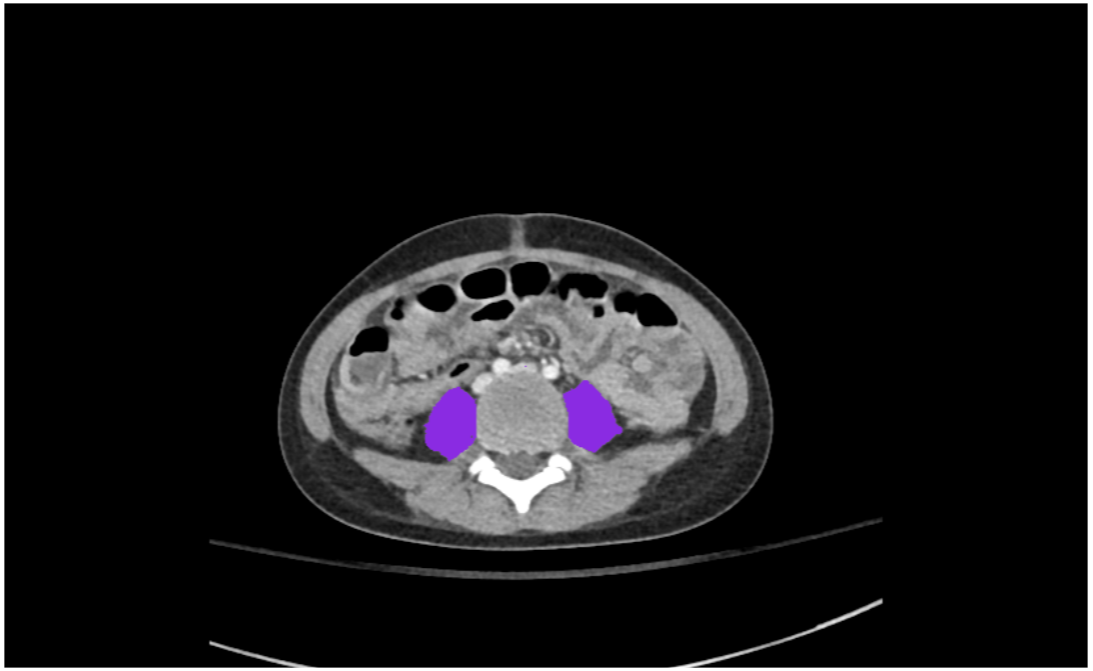
An example of a semi-automatically drawn region-of-interest (ROI) to measure total psoas muscle area (tPMA).

Abdominal CT images were acquired at 70–100 kVp according to patient weight (0–15 kg: 70 kVp, 15–40 kg: 80 kVp, >40 kg: 100 kVp) with automatic tube current modulation during the portal phase (45–60 s from contrast injection). After determining the L3–4 and L4–5 levels in reference to reformatted sagittal images, tPMA was retrospectively measured using commercial software, Aquarius iNtuition Viewer (ver. 4.4.11, Tera-Recon Inc., Foster City, CA, USA), which is commonly utilized in various studies for segmentation tasks, particularly in the evaluation of tPMA, through semi-automated segmentation with expert supervision (17, 18). Psoas muscles were extracted by applying Hounsfield unit thresholds between −29 and +150. Following the automatic segmentation, an experienced image analyzer, blinded to the medical records, reviewed and corrected the ROIs to ensure accuracy according to standardized procedures. tPMA was calculated as the sum of the left and right psoas muscle areas at the level of the L3–4 and L4–5 intervertebral discs (Figure 1).

### 2.3 Statistical analysis

Statistical analyses were performed using R software (ver. 4.3.1, R Foundation for Statistical Computing, Vienna, Austria). Continuous variables are presented as median (interquartile range, IQR). Standard growth charts for tPMA according to sex were obtained using quantile regression with smoothed polynomials using the R package “quantreg” version 5.99.1. The *z*-score was calculated to represent the relative distribution of the patient's tPMA value within the same age and gender group, based on the 10th, 50th, and 90th percentiles predicted by LOESS. The estimated 50th percentile tPMA values were compared with the Wilcoxon rank sum test or Wilcoxon signed rank test based on the measured level and sex. Graphs for weight and height according to sex and age were created. The 50th percentile weight and height of our study population by sex were compared with the 50th percentile weight and height derived from the Korean national growth chart provided by the Korea National Health and Nutrition Examination Survey (KNHANES) 2017 using the Wilcoxon rank sum test to assess the representativeness of the study population as healthy Korean children (19). Pearson's correlation coefficients were calculated to evaluate correlations between tPMA values at the L3–4 and L4–5 levels, tPMA and weight, as well as tPMA and height for each sex. *P* values less than 0.05 were considered statistically significant.

## 3 Results

### 3.1 Comparison of weight and height growth charts with data from KNHANES 2017

**Figure 2:**
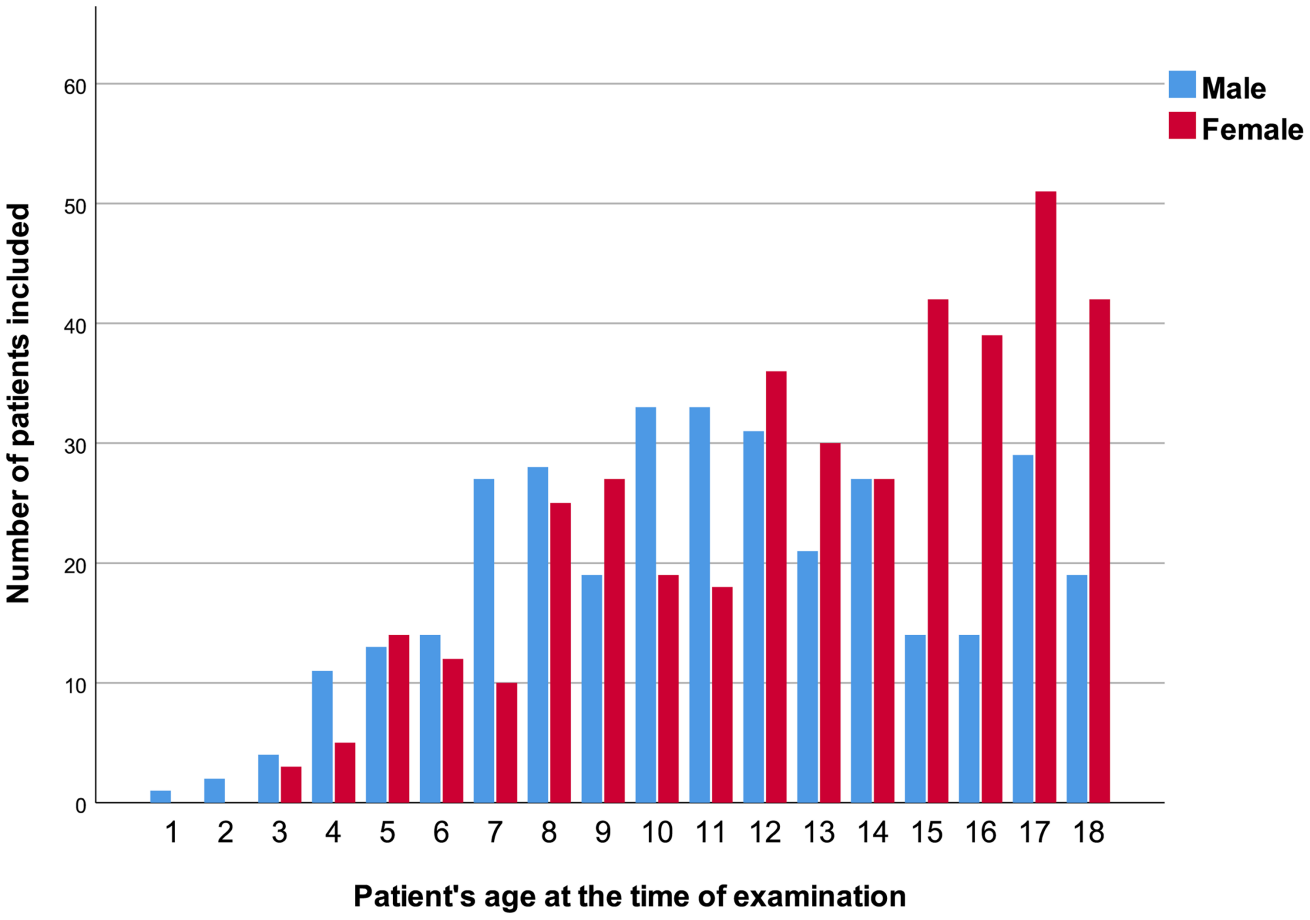
Number of patients according to age at the time of examination.

A total of 740 patients were enrolled [M:F = 340:400, 12 years (IQR: 9–16)] from an initial 764 patients, with two patients excluded due to image measurement errors and 22 patients excluded due to weight and height data missing from medical records. All included patients were ethnically Korean children. The mean age of the female patients was significantly older than the male patients [males: 11 years [IQR: 8–14], females: 14 years [IQR: 10–16], *P* < 0.001], with the included patients ranging in age from 1 to 18 years for males and 3–18 years for females. Only one-year-old male and two-year-old males were included, while none of the females included in this study were younger than two years old (Figure 2).

**Figure 3:**
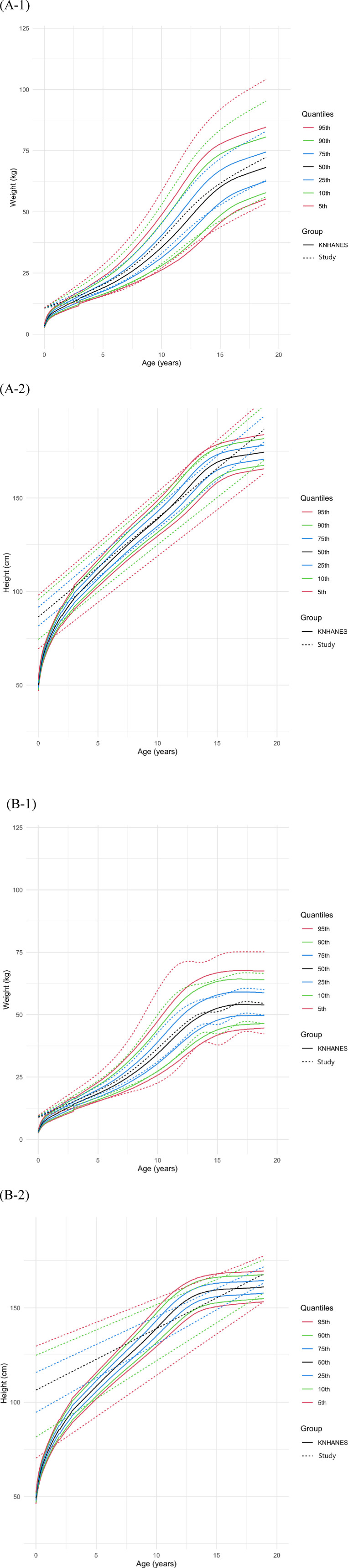
Comparison of weight and height growth charts in males **(A)** and females **(B)** that were drawn using data from the Korea national health and nutrition examination survey (KNHANES) 2017.

Weight and height growth charts were generated for males and females (Figure 3) separately, with the 5th to 95th percentiles compared to the KNHANES 2017 cohort. The 50th percentile height of females in our study population was not significantly different from that of the KNHANES population [study: 137.2 cm [IQR: 121.8–152.6], KNHANES: 135.6 cm [IQR: 106.7–158.6], *P* = 0.077]. There was no significant difference in weight between the female populations [study: 34.1 kg [IQR: 18.9–51.0], KNHANES: 32.1 kg [IQR: 17.8–51.0], *P* = 0.348]. In males, there were no significant differences in the 50th percentile weight [study: 36.0 kg [IQR: 19.6–58.8], KNHANES: 33.2 kg [IQR: 18.4–56.8], *P* = 0.164] or height [study: 136.5 cm [IQR: 111.5–161.6], KNHANES: 135.9 cm [IQR: 107.85–165.9] *P* = 0.434] when our study population was compared to the KNHANES population.

### 3.2 Sex-specific growth chart of tPMA

**Figure 4:**
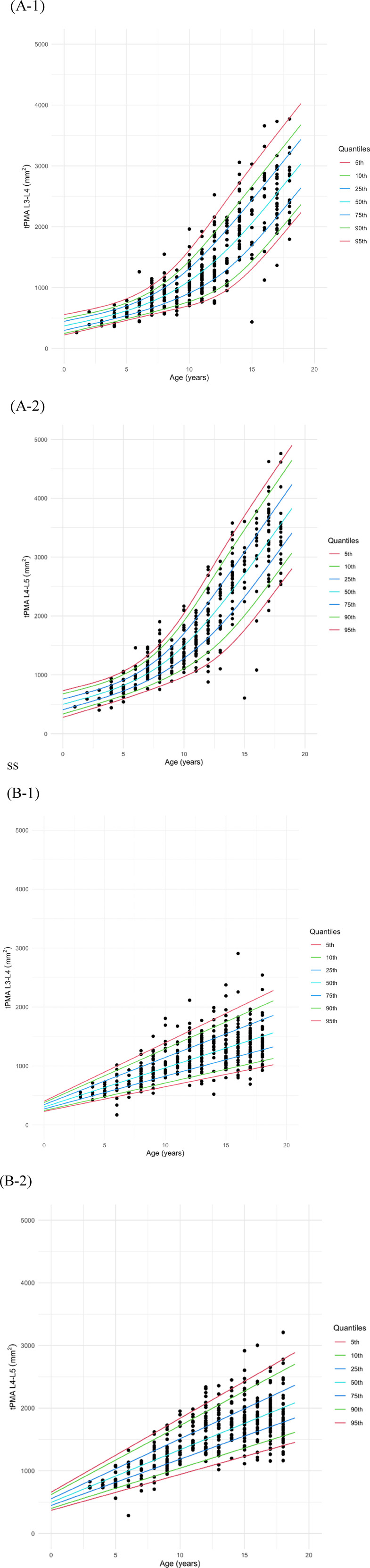
Growth charts of total psoas muscle area (tPMA) in males **(A)** and females **(B)** at the L3–4 and L4–5 levels.

Growth charts of tPMA, with the 5th to 95th percentiles displayed, were generated for males (Figure 4A) and females (Figure 4B) separately at both the L3–4 and L4–5 levels. To validate the goodness-of-fit of the generated growth charts, the percentage of measurements below the 5th percentile or above the 95th percentile was calculated. For both male and female, at the L3–4 and L4–5 levels, the percentages ranged between 4% and 6%, confirming that the model was well-fitted. The 50th percentile tPMA values were consistently larger at the L4–5 level compared to the L3–4 level for both sexes across all ages (*P* < 0.001, both). tPMA at the L3–4 and L4–5 levels demonstrated significant positive correlations (*r* = 0.948, *P* < 0.001).

At the L3–4 level, the 50th percentile tPMA values ranged from 416 to 2,802 mm^2^ in males and 370–1,501 mm^2^ in females. Males exhibited larger 50th percentile tPMA values at the L3–4 level across all ages [males: 1,037 mm^2^ [IQR: 604–1,876], females: 933 mm^2^ [IQR: 618–1,247], *P* < 0.001], except for the age range of 3 years 5 months to 6 years 10 months. The difference in tPMA values between the two sexes was less than 16 mm^2^ for this specific age range and was not significant (*P* = 0.501). The difference between males and females increased with age at the L3–4 level.

The 50th percentile tPMA values at L4–5 ranged from 556 to 3,563 mm^2^ in males and 579–2,007 mm^2^ in females. Similarly, the 50th percentile tPMA values at the L4–5 level were larger in males across all ages [males:1,391 mm^2^ [IQR: 798–2,487], females: 1,290 mm^2^ [IQR: 893–1,687], *P* = 0.008] than females, except for the age range of 2 months to 8 years 2 months. Between 2 months and 8 years 2 months, the 50th percentile tPMA values at the L4–5 level were significantly larger in females than in males, with a difference of less than 96 mm^2^ [males: 754 mm^2^ [IQR: 623–930], females: 845 mm^2^ [IQR: 677–1,013], *P* = 0.030]. At the L4–5 level, the difference between males and females increased with age.

**Figure 5:**
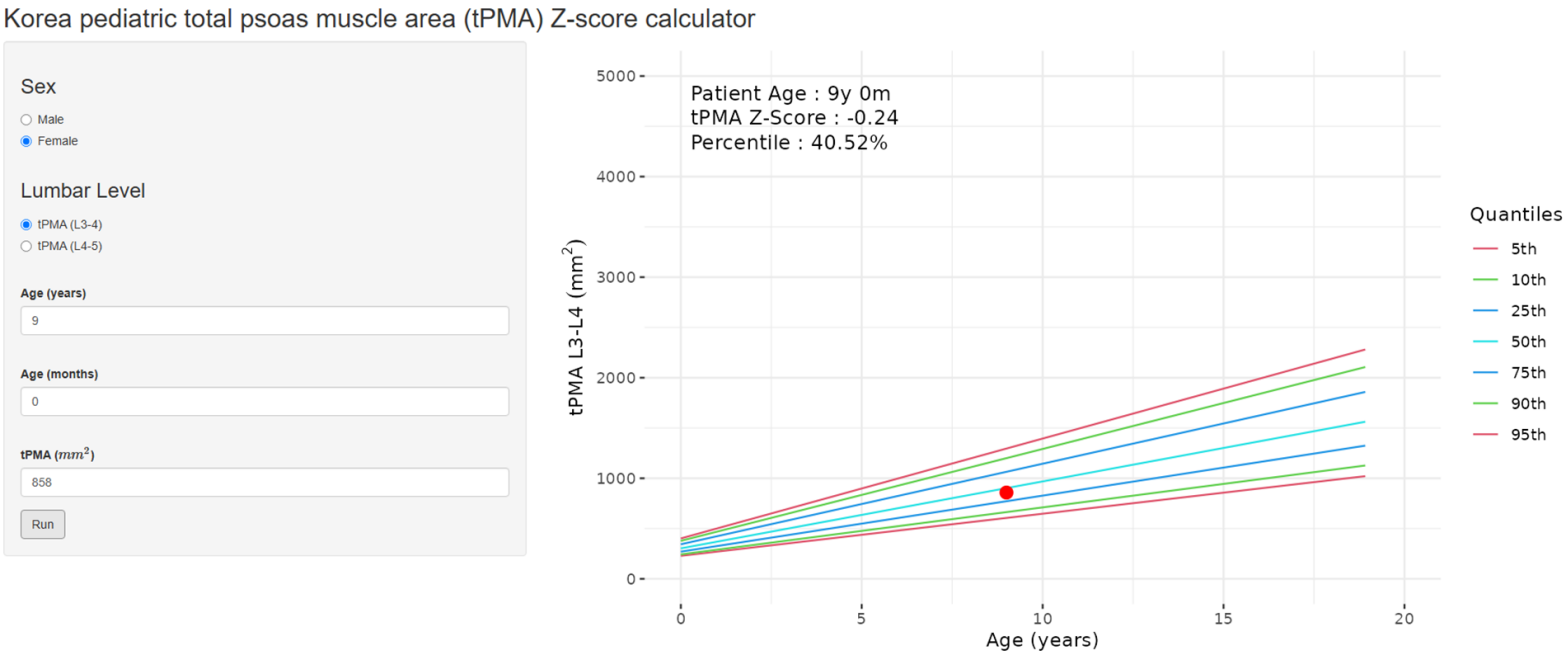
An example of the online calculator (https://pediatricpmakorea.shinyapps.io/kPMA) being applied to a 9-year-old female to calculate total psoas muscle area (tPMA) at L3–4 intervertebral disc level, 8,580 mm^2^ and a *z*-score −0.24 and percentile of 40.52.

To facilitate the easy calculation of *z*-scores and percentiles of tPMA according to sex and age for patients under 18 years old, an online calculator (https://pediatricpmakorea.shinyapps.io/kPMA/) was developed (Figure 5).

### 3.3 Correlation of tPMA with weight and height

**Figure 6:**
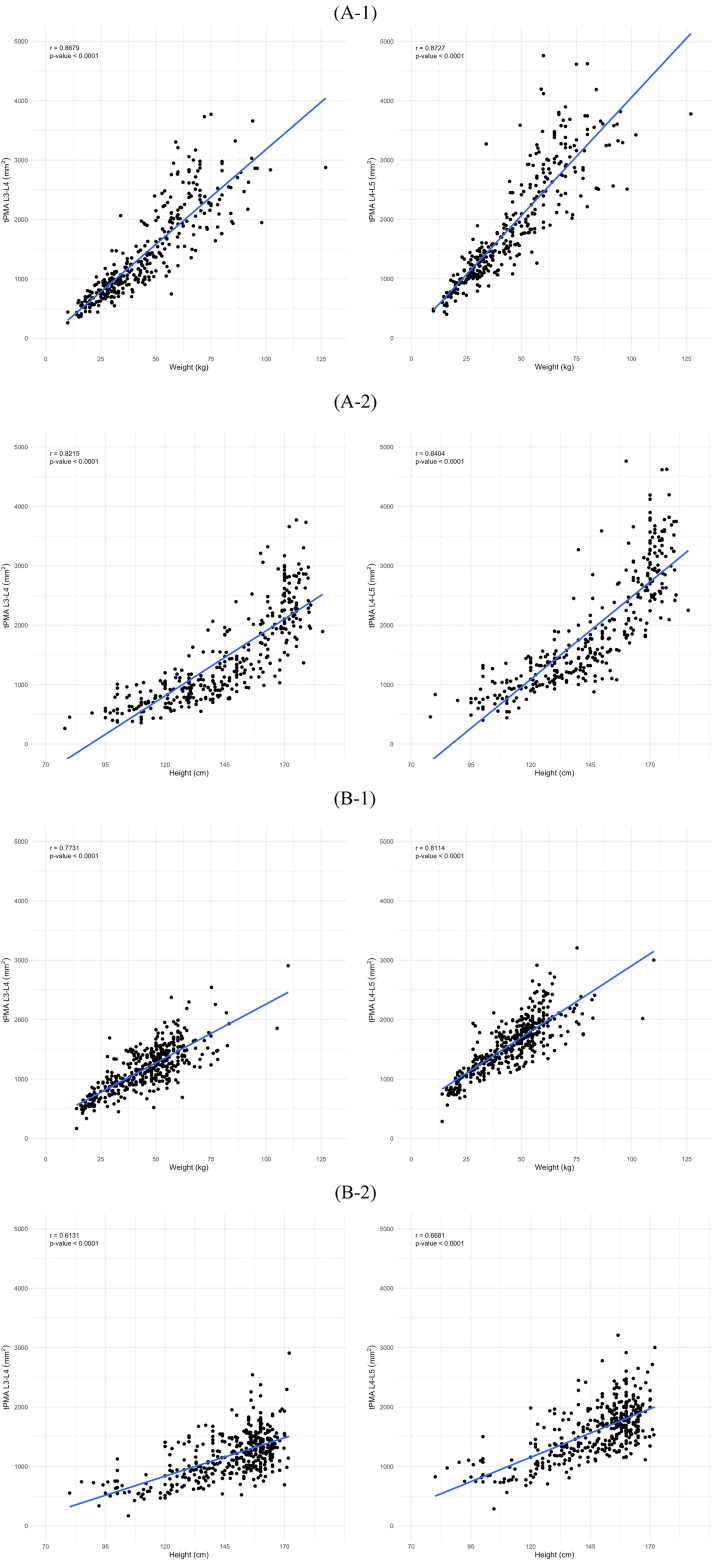
Correlation of total psoas muscle area (tPMA) with weight and height in males **(A)** and females **(B)** at the L3–4 and L4–5 levels. **(A-1)** Correlation between tPMA and weight in males at the L3–4 and L4–5 levels. **(A-2)** Correlation between tPMA and height in males at the L3–4 and L4–5 levels. **(B-1)** Correlation between tPMA and weight in females at the L3–4 and L4–5 levels. **(B-2)** Correlation between tPMA and height in females at the L3–4 and L4–5 levels.

In males, tPMA at the L3–4 and L4–5 levels demonstrated significant positive correlations with weight (L3–4 *r* = 0.87, *P* < 0.001; L4–5 *r* = 0.87, *P* < 0.001) and height (L3–4 *r* = 0.82, *P* < 0.001; L4–5 *r* = 0.84, *P* < 0.001) (Figure 6). Similarly, in females, both tPMA at the L3–4 and L4–5 levels also showed significant positive correlations with weight (L3–4 *r* = 0.77, *P* < 0.001; L4–5 *r* = 0.81, *P* < 0.001) and height (L3–4 *r* = 0.61, *P* < 0.001; L4–5 *r* = 0.67, *P* < 0.001) (Figure 6).

## 4 Discussion

We proposed sex-specific and age-specific reference values for tPMA at the intervertebral disc levels of L3–4 and L4–5 for children between the ages of 1 and 18 years based on a population that was not statistically different from the KNHANES 2017 cohort. Additionally, we developed an online calculator that can generate tPMA percentiles and z-scores with the input of sex and age, strengthening the practical utility of our tPMA measurements.

Sarcopenia is generally defined as the natural aging-related loss of skeletal muscle mass and function (1). Even though sarcopenia and its impact have received little attention in pediatric settings, previous reports have shown that children with end-stage liver disease, inflammatory bowel disease, and kidney failure, have lower tPMA on CT compared to matched healthy controls. Although BMI and other anthropomorphic metrics are simple to calculate, they fail to correctly evaluate body composition and do not correlate with tPMA. tPMA can be an important independent representative of nutritional status and a predictor of sarcopenia in adults as well as children (20, 21). Also, previous pilot studies associated sarcopenia with prolonged hospital stays and increased post-treatment complications in pediatric patients suffering from leukemia, inflammatory bowel disease or chronic liver disease (3–5, 22). However, determining normal ranges for tPMA in the pediatric population has been challenging because muscle mass changes greatly with growth (6–8). CT-based tPMA values at various intervertebral disc levels have been published for adults and adolescents including the L2–3, L3, L3–4 and L4–5 levels (21, 23–25). However, standard values are assumed to differ by race and it is necessary to incorporate a specific standardized normal tPMA value to conduct customized sarcopenia research in Korea. Reference values for each candidate's vertebral level are required to assess a consistent site in each study. Therefore, we measured the tPMA at the two levels that are most likely to be studied. We evaluated the psoas muscle at two intervertebral disc levels, L3–4 and L4–5 because we expect tPMA values measured at these intervertebral disc levels to be more precise than those measured at other locations. Measuring at L3–4 and L4–5 may help reduce the possibility of location errors compared to measuring tPMA at the level of the vertebral body. Measurements at other intervertebral levels, such as L5-S1, could potentially yield less reliable results due to changes in muscle positioning and pelvic alignment. Also, since tPMA values at the L3–4 and L4–5 levels were positively correlated with significance in this study, tPMA values at the L3–4 level can be acquired and evaluated even with a chest CT if we extend the field of view.

Lurz et al. reported reference values for tPMA at the L3–4 and L4–5 levels in Canadian children, based on 779 patients (M:F = 499:280) aged 1–16 (6). Reference values for tPMA have also been reported for an American population of 782 patients (M:F = 494:288) aged 1–17 and a Japanese population of 593 patients (M:F = 335:258) aged 1–17, but these reference values were based on measurements taken only at the L3–4 level (8, 17). We proposed sex-specific and age-specific reference values for tPMA at the L3–4 and L4–5 levels, based on the data of 740 patients (M:F = 340:400) aged 1–18. Still, the three previous studies commonly found tPMA to continuously increase with age, with significant differences between males and females appearing at approximately age 10, from which male patients show a steeper climbing curve. Our study also demonstrated a similar trend, with increased differences between males and females from the age of 9 years and 5 months at the L3–4 level and from the age of 9 years and 6 months at the L4–5 level.

Comparing the 50th percentile of commonly measured tPMA values at the L3–4 level, the American population (1–17 years old) had tPMA values that ranged from 342 to 3,051 mm^2^ in males and 305 to 1,877 mm^2^ in females, while the Canadian population (1–16 years old) had values that ranged from 394 to 3,050 mm^2^ in males and 365 to 2,336 mm^2^ in females (6, 7). In contrast, our results (1–18 years old) showed tPMA ranging from 416 to 2,802 mm^2^ in males and 370 to 1,501 mm^2^ in females. The tPMA differences between our study population and the American and Canadian populations were small at younger ages with 416 mm^2^ for males (342 mm^2^ for Americans, 394 mm^2^ for Canadians) and 370 mm^2^ for females (305 mm^2^ for Americans, 365 mm^2^ for Canadians) at 1 year of age. However, tPMA demonstrated a relatively gradual growth curve as children grew older, with tPMA at the age of greatest measurement in each population being 2,802 mm^2^ for males (3,051 mm^2^ for Americans, 3,050 mm^2^ for Canadians) and 1,501 mm^2^ for females in our study population (1,877 mm^2^ for Americans, 2,336 mm^2^ for Canadian). More specifically, in comparison to the American population, both males and females in our study exhibited larger tPMA between the ages 1 and 3. However, as age increased, the tPMA of our study population also gradually increased, resulting in smaller tPMA compared to the American population.

For the Japanese population (1–17 years old), the 50th percentile tPMA ranged from 380 to 2,320 mm^2^ in males and 340–1,490 mm^2^ in females (8), and in the same age range of our study population, ranged from 416 to 2,550 mm^2^ and 370–1,434 mm^2^, respectively, which was the most similar result among the reports from other countries. When compared to the Japanese population, males in our study consistently exhibited larger tPMA, with differences increasing up to 230 mm^2^ as they grew older until 17 years old. On the other hand, females in our study showed relatively smaller tPMA since the age of 9.

For tPMA at the L4–5 level, only the study with the Canadian population provided reference values of 498–3,513 mm^2^ for males and 447–2,704 mm^2^ for females (6). In our study population at the same age (1–16 years), tPMA ranged from 556 to 2,989 mm^2^ in males and 579–1,839 mm^2^ in females, similar to trends observed for the tPMA at the L3–4 level. Our study population demonstrated larger tPMA in younger ages, with a gradual increase eventually resulting in smaller tPMA compared to the Canadian population as the children grew older. This difference is thought to arise from variations in race proportions within each population and therefore suggests that when data from countries with a large number of Caucasian children, such as Canada and the US are used as a reference to evaluate Asians, there is the possibility that sarcopenia may be overdiagnosed (26). For example, in our study population, the 50 percentile tPMA values at L3–4 and L4–5 for 10-year-old males were 1,111 mm^2^ and 1,494 mm^2^, respectively. When data from the Canadian population of the same sex and age were analyzed, the corresponding percentiles for these tPMA values were 9.7 and 21.8 for males and females, respectively.

Our study results are consistent with previous data in which tPMA obtained from CT cross-sections correlated strongly with weight and height (Figure 6). This was also reported previously for the Japanese (8) and large Canadian cohort (6). Unlike the Japanese study where height and weight data was missing for about 20% of cases, our study population had more complete medical records and our findings seem more robust since we provided the full height and weight information.

Thus, our study has the advantage of including both L3–4 and L4–5 tPMA values, compared to other studies. Furthermore, because the maximum age range for enrollment is up to 18, our data probably provide better reference values when studying sarcopenia in Asian pediatric, growing adolescent, and young populations. Additionally, by developing an online calculator as a reference guide, this data can be easily accessible to researchers.

This study has several limitations. Firstly, it was a retrospective, single-center study conducted in a tertiary healthcare center. We aimed to enroll patients without underlying diseases or any growth or nutritional issues at the time of examination. Children coming to a tertiary hospital may not necessarily be considered completely healthy. However, recruiting completely healthy pediatric volunteers for CT examinations, which involve radiation exposure, is not considered ethically feasible. Therefore, we opted to compare our findings with the KNHANES 2017 cohort, a study conducted with a normal population. Secondly, not many of our patients were less than three years of age even though we recruited a substantial number of patients. This is because CT is not the primary routine modality for screening abdominal pain in children this young, so there might be a sample bias for patients who are not yet three years old. Third, our female population was significantly taller compared to the KNHANES population. This may be due to growth differences for different periods. Finally, our study population showed similar tPMA ranges to the Japanese population. However, growth patterns may differ depending on lifestyles even within the same race. Our study revealed differences in tPMA, with males in our study population having larger tPMA and older female patients showing smaller tPMA compared to the Japanese population.

In conclusion, we provide age-specific and sex-specific growth charts for tPMA at the L3–4 and L4–5 intervertebral disc levels, which can serve as a guide for evaluating muscle mass in Korean children and adolescents under 18 years old.

## Data Availability

The original contributions presented in the study are included in the article/Supplementary Material, further inquiries can be directed to the corresponding author.
